# Comprehensive analysis of RNA binding motif protein 3 (RBM3) in non‐small cell lung cancer

**DOI:** 10.1002/cam4.3149

**Published:** 2020-06-03

**Authors:** Annette Salomonsson, Patrick Micke, Johanna S. M. Mattsson, Linnea La Fleur, Johan Isaksson, Mats Jönsson, Björn Nodin, Johan Botling, Mathias Uhlén, Karin Jirström, Johan Staaf, Maria Planck, Hans Brunnström

**Affiliations:** ^1^ Department of Clinical Sciences Division of Oncology and Pathology Lund University Lund Sweden; ^2^ Department of Immunology, Genetics and Pathology Uppsala University Uppsala University Hospital Uppsala Sweden; ^3^ Department of Respiratory Medicine Gävle Hospital Gävle Sweden; ^4^ Centre for Research and Development Uppsala university/County Council of Gävleborg Gävle Sweden; ^5^ Department of Clinical Sciences Division of Oncology and Pathology Lund University Lund Sweden; ^6^ Science for Life Laboratory Royal Institute of Technology Stockholm Sweden; ^7^ School of Biotechnology AlbaNova University Center Royal Institute of Technology Stockholm Sweden; ^8^ Department of Genetics and Pathology Laboratory Medicine Region Skåne Lund Sweden; ^9^ Department of Respiratory medicine and Allergology Skåne University Hospital Lund Sweden

**Keywords:** biomarker, gene expression, immunohistochemistry, survival, tissue microarray

## Abstract

**Aims:**

High expression of the RNA‐binding motif protein 3 (RBM3) correlates with improved prognosis in several major types of cancer. The aim of the present study was to examine the prognostic value of RBM3 protein and mRNA expression in non‐small cell lung cancer (NSCLC).

**Methods and results:**

Immunohistochemical expression of RBM3 was evaluated in surgically treated NSCLC from two independent patient populations (n = 213 and n = 306). Staining patterns were correlated with clinicopathological parameters, overall survival (OS), and recurrence‐free interval (RFI). Cases with high nuclear RBM3 protein expression had a prolonged 5‐year OS in both cohorts when analyzing adenocarcinomas separately (*P* = .02 and *P* = .01). RBM3 remained an independent prognostic factor for OS in multivariable analysis of cohort I (HR 0.44, 95% CI 0.21‐0.90) and for RFI in cohort II (HR 0.38, 95% CI 0.22‐0.74). In squamous cell carcinoma, there was instead an insignificant association to poor prognosis. Also, the expression levels of *RBM3* mRNA were investigated in 2087 lung adenocarcinomas and 899 squamous cell carcinomas assembled from 13 and 8 public gene expression microarray datasets, respectively. The *RBM3* mRNA levels were not clearly associated with patient outcome in either adenocarcinomas or squamous cell carcinomas.

**Conclusions:**

The results from this study support that high protein expression of RBM3 is linked to improved outcome in lung adenocarcinoma.

## Graphical abstract

High protein expression of RBM3 was associated with longer survival time in resected lung adenocarinoma in two independant cohorts. The association to outcome was not seen in squamous cell carcinomas or for mRNA levels.

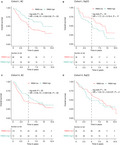

## 1 INTRODUCTION

Due to high incidence and poor prognosis, lung cancer is the most common cause of cancer death worldwide.^1^ Most lung cancers are non‐small cell lung cancer (NSCLC), mainly constituted of the histological subgroups adenocarcinoma (AC) and squamous cell carcinoma (SqCC). Patients with early stage disease can be treated surgically, with or without adjuvant chemo‐ and radiotherapy, and have a better chance of long‐term survival. However, due to high recurrence rate, the 5‐year survival after curatively intended surgery is still poor, with only a limited effect from adjuvant treatment.^2^ Hence, there is a need for novel prognostic and treatment predictive biomarkers for improved treatment and follow‐up of these patients.

RNA‐binding motif protein 3 (RBM3) binds to RNA and is thereby involved in the regulation of gene expression.^3^ High RBM3 protein expression has been reported as a favorable prognostic marker in several types of cancer,^4^, ^5^, ^6^, ^7^, ^8^, ^9^, ^10^, ^11^, ^12^, ^13^, ^14^ recently also in NSCLC.^15^ The prognostic role of *RBM3* mRNA expression and the correlation between RBM3 protein and mRNA expression have been less well studied.^5^, ^16^

The aim of this study was to examine the expression of RBM3 at the protein and mRNA levels in NSCLC and to correlate the results with patient outcome.

## 2 MATERIAL AND METHODS

### 2.1 Patient material and characteristics

The study was conducted on two cohorts. The first cohort (cohort I) was based on the “Southern Swedish Lung Cancer Study”.^17^ This prospective study non‐selectively included patients with primary lung cancer who underwent surgical treatment at the Skåne University Hospital, Lund, Sweden, in 2005‐2011. The second cohort (cohort II) was based on the "Uppsala NSCLC II cohort”. This retrospective study included consecutive samples of primary NSCLC surgically treated at the University Hospital in Uppsala, Sweden, in 2006‐2010.^18^, ^19^ The present study included 213 cases in cohort I (131 AC, 69 SqCC, two adenosquamous cell carcinomas, eight large cell neuroendocrine carcinomas, one large cell carcinoma, and two sarcomatoid carcinomas) and 306 cases in cohort II (194 AC, 91 SqCC, five adenosquamous cell carcinomas, nine large cell neuroendocrine carcinomas, five large cell carcinomas, and two sarcomatoid carcinomas). Only patients that were surgically treated for primary NSCLC tumors with no neoadjuvant treatment or chemotherapy for another malignancy six months before surgery were included in the present study. In cohort II, evaluation of RBM3 protein expression was possible in 36 paired metastases (showing similar staining patterns), see Data S1. Mutation status for 82 genes was available for 297 of the cases with evaluable RBM3 in cohort II.^19^

All histopathological slides for the cases were previously reviewed^17^, ^18^ and the diagnoses updated in accordance with the 2015 WHO classification and TNM 7.^20^, ^21^, ^22^ All changes compared to original diagnoses were confirmed by two pathologists (HB and PM). For AC, growth pattern was evaluated (HB) and the cases were stratified into three groups: minimally invasive AC/predominant lepidic AC, predominant acinary/papillary AC, and mucinous or predominant micropapillary/solid AC, respectively.

For 5‐year overall survival (OS) analysis, data were gathered from the Swedish Cancer Registry. The registry was consulted on 26 June 2018 for cohort I, and on 29 March 2019 for cohort II.

For analysis of recurrence‐free interval (RFI), patients were followed until recurrence or until their final/latest oncological follow‐up visit. The last check‐up in the medical records was in February 2019 for cohort I, and in January 2016 for cohort II. Patients who were diagnosed with early recurrences (within 90 days after surgery), patients who never had any follow‐up visits, and patients with metastatic disease at time of surgery were excluded from subsequent RFI analysis. If emigration occurred before recurrence or final check‐up, patients were censored at the date of the last oncologic follow‐up. If death occurred before recurrence or final check‐up, patients were censored at the date of the last oncologic follow‐up, except for four cases in cohort II which were censored at the date of death due to difficulties in retrieving medical records. If metastatic disease (other than the lung cancer studied) occurred before lung cancer recurrence or final check‐up, patients were censored at the date of diagnosis of the new tumor.

The study was conducted in adherence with the Declaration of Helsinki and approved by the regional ethical review boards in Lund (Dnr 2004/762 and 2008/702) and Uppsala (Dnr 2012/532), respectively.

### 2.2 Immunohistochemistry and staining evaluation

For immunohistochemistry (IHC) analysis, 4‐μm thick sections from tissue microarrays (TMAs) were used. The TMAs had three (cohort I) or two (cohort II) cores, 1 mm in diameter, from each case. In all cases there were at least 200 viable tumor cells (in the vast majority of cases, more than 1000 viable cells). The tissue sections were automatically pre‐treated using the PT Link system (DAKO, buffer pH 7, RT 30 minutes) and then stained in an Autostainer Plus (DAKO; Copenhagen, Denmark). For RBM3 staining, we used the mouse monoclonal anti‐RBM3 antibody AMAb90655 clone CL0296 (Atlas Antibodies AB, Stockholm, Sweden, diluted 1:100). The specificity of the antibody had been previously validated.^5^ For Ki67 staining we used the mouse monoclonal anti‐Ki67 clone MIB‐1 (DAKO; Copenhagen, Denmark, diluted 1:200). In addition to microscopic evaluation, the slides were scanned and evaluated using the pathXL software (Philips, Amsterdam, The Netherlands).

The RBM3 and Ki67 stainings were evaluated by two independent observers (AS and HB for RBM3, AS and MJ for Ki67) who were blinded to clinical data. Scoring differences were discussed between the evaluators for consensus.

For RBM3, the fraction of viable tumor cells expressing RBM3 in the nucleus (nuclear fraction, NF) was scored as 0 (0%‐1%), 1 (>1%‐25%), 2 (>25%‐50%), 3 (>50%‐75%) or 4 (>75%). The nuclear staining intensity (NI) was scored as 0 (negative), 1 (weak), 2 (moderate) or 3 (strong). In case of varying nuclear fraction or intensity between the cores within a sample, the dominating pattern was denoted. A combined nuclear score (NS) was constructed by multiplying nuclear fraction (NF) and nuclear intensity (NI), thus ranging from 0 to 12.

Cohort I was used as a discovery cohort for identification of an optimal cut‐off for classifying RBM3 low vs high samples, and cohort II was used as a validation cohort. Kaplan‐Meier plots with log‐rank test were used to test different cut‐offs of NS (0‐12). Prognostic analyses were performed on AC and SqCC separately and did not include any other histologies since the latter were too few for subgroup analyses. For AC, dichotomization based on NS ≥6 and ≥8 produced similar results, and since the cut‐off ≥8 was considered more clinically manageable, AC samples with NS ≥8 were classified as having a high RBM3 protein expression. For SqCC, dichotomization based on NS ≥4 produced the best separation of groups and was therefore chosen as cut‐off for SqCC samples.

For evaluation of the Ki67 staining, the fraction of viable tumor cells expressing Ki67 in the nucleus was scored as 0 (0%‐1%), 1 (>1%‐10%), 2 (>10%‐25%), 3 (>25%‐50%), 4 (>50%‐75%), or 5 (>75%). In case of varying nuclear fractions between the cores within a sample, the core with the highest fraction was denoted.

### 2.3 RBM3 gene expression in cohort II

For cohort II, fragments per kilobase per million (FPKM) counts generated through RNA sequencing (RNAseq) were available for 175 patients (104 AC, 58 SqCC and 13 cases of other histology), of which 19 samples (10 AC, 7 SqCC and two cases of other histology) also had data from paired normal tissue.

RNAseq analysis was performed as described previously.^23^ Briefly, RNA was extracted from frozen tissue samples, and prepared using the Illumina TruSeq RNA Sample Prep Kitv2 (Illumina, San Diego, CA, USA) with polyA selection. The sequencing was performed on the Illumina HiSeq2500 (Illumina), using the standard Illumina RNAseq protocol with a read length of 2 × 100 bp. The raw sequencing data have been deposited at http://www.ncbi.nlm.nih.gov/geo/ with the accession number GSE81089. We tested two different cut‐offs for classifying samples as having high *RBM3* gene expression levels. The samples were divided into two groups with either the upper ½ of the samples or the upper ⅓ of the samples classified as high, based on gene expression levels.

### 2.4 Prognostic analysis of RBM3 gene expression

The association of *RBM3* gene expression with patient outcome was analyzed as described^24^ in 2087 lung AC and 899 SqCC assembled from 13^25^, ^26^, ^27^, ^28^, ^29^, ^30^, ^31^, ^32^, ^33^, ^34^, ^35^, ^36^, ^37^ and 8^26^, ^27^, ^31^, ^34^, ^35^, ^36^, ^37^, ^38^ public gene expression microarray cohorts, respectively. Briefly, for each microarray cohort, gene expression profiles were normalized and the *RBM3* probe/probe set with the highest standard deviation in expression was identified and subsequently mean‐centered across all cases in the cohort. For each cohort, we tested two different cut‐offs for classifying samples as having high *RBM3* gene expression levels, that is, the upper ½ of the samples or the upper ⅓ of the samples classified as high, based on gene expression levels. For cohorts including both AC and SqCC, this analysis was performed for each histological type separately. Patient outcome was censored at 5 years due to different follow‐up times between the cohorts.

### 2.5 Statistical analysis

Kruskal‐Wallis test, Fisher's exact test and Mann‐Whitney U test/Wilcoxon rank‐sum test were used for comparisons of demographic and pathological data between groups. Kaplan‐Meier curves with log‐rank test were used for survival probabilities and cumulative RFI. Univariable and multivariable Cox proportional hazards regression models were used to further compare groups and to generate hazard ratio (HR) and 95% confidence interval (CI). Multivariable models were adjusted for age, gender, stage (I, II, III, and IV), growth pattern (AC only), smoking (current, past, and never), adjuvant therapy, and patients’ performance status (the latter available for cohort II only).^39^

A *P*‐value < .05 was considered statistically significant. All statistical analyses were performed with both the R software, version 3.4.2^40^ and MedCalc 14.12.0 (MedCalc Software bvba, Ostend, Belgium).

## 3 RESULTS

### 3.1 Comparison of the two cohorts

Patient characteristics and clinicopathological data for AC and SqCC cases in cohorts I and II are summarized in Table 1.

**Table 1:** Patient and tumor characteristics

Histology	AC		SqCC	
Cohort	Cohort I (n = 131)	Cohort II (n = 194)	Cohort I (n = 69)	Cohort II (n = 91)
Age at surgery, median (range)	68 y (50‐83 y)	67 y (42‐83 y)	72 y (48‐85 y)	68 y (46‐84 y)
Follow‐up time, OS, median (range)	6.0 y (12 d – 12.6 y)	4.8 y (3 d – 13.2 y)	6.8 y (98 d – 12.4 y)	7.3 y (5 d – 13.2 y)
Gender, women	84 (64%)	107 (55%)	32 (46%)	38 (42%)
Stage				
I	67 (51%)	120 (62%)	32 (46%)	60 (66%)
II	35 (27%)	32 (16%)	24 (35%)	25 (27%)
III	25 (19%)	35 (18%)	10 (14%)	6 (7%)
IV	4 (3%)	7 (4%)	3 (4%)	0 (0%)
T stage				
T1	50 (38%)	98 (51%)	14 (20%)	36 (40%)
T2	52 (40%)	70 (36%)	37 (54%)	41 (45%)
T3	23 (18%)	21 (11%)	15 (22%)	11 (12%)
T4	6 (5%)	5 (3%)	3 (4%)	3 (3%)
N stage				
N0	92 (70%)	143 (74%)	50 (72%)	77 (85%)
N1	25 (19%)	22 (11%)	15 (22%)	11 (12%)
N2	14 (11%)	29 (15%)	4 (6%)	3 (3%)
AC growth pattern				
Minimally invasive/ lepidic	4 (3%)	11 (6%)	—	—
Acinary/ papillary	93 (72%)	118 (63%)	—	—
Micropapillary/ solid/ mucinous	33 (25%)	58 (31%)	—	—
Not determinable	1	6	—	—
Smoking				
Current	72 (55%)	94 (48%)	38 (55%)	52 (57%)
Former (>1 year)	43 (33%)	78 (40%)	30 (43%)	34 (37%)
Never	16 (12%)	22 (11%)	1 (1%)	5 (5%)
Adjuvant treatment				
Yes	53 (43%)	72 (41%)	27 (43%)	38 (44%)
No	71 (57%)	103 (59%)	36 (57%)	48 (56%)
Missing data	7	19	6	5
Recurrence				
Yes	46 (38%)	71 (43%)	21 (34%)	30 (37%)
No	76 (62%)	93 (57%)	41 (66%)	51 (63%)
Missing data	9	30	7	10
RBM3 IHC classification				
High	42 (33%)	73 (38%)	32 (47%)	35 (39%)
Low	86 (67%)	117 (62%)	36 (53%)	55 (61%)
Missing data	3	4	1	1

Abbreviations: AC, adenocarcinoma; d, days; IHC, immunohistochemical; NS, nuclear score; OS, overall survival; SqCC, squamous cell carcinoma; y, years.

For cohort II, the SqCC cases had lower stage, lower T stage and younger age, and the AC cases had lower T stage, compared to cohort I (Mann‐Whitney test, all *P* ≤ .03). Apart from these findings, there were no other differences between cohorts I and II for AC or SqCC, respectively, regarding the number of cases with high/low RBM3 expression, stage (TNM and separate T and N stages), growth pattern (AC only), age, gender, smoking, number of cases receiving adjuvant treatment, number of deaths at 5 years, or number of recurrences (Kruskal‐Wallis test, Fisher's test, and Mann‐Whitney test). Also, there was no significant difference in frequency of EGFR mutations, 5 (10%) of 52 tested AC cases in cohort I vs 25 (13%) of 194 AC in cohort II.

### 3.2 RBM3 protein expression and association to clinicopathological data

**Figure 1:**
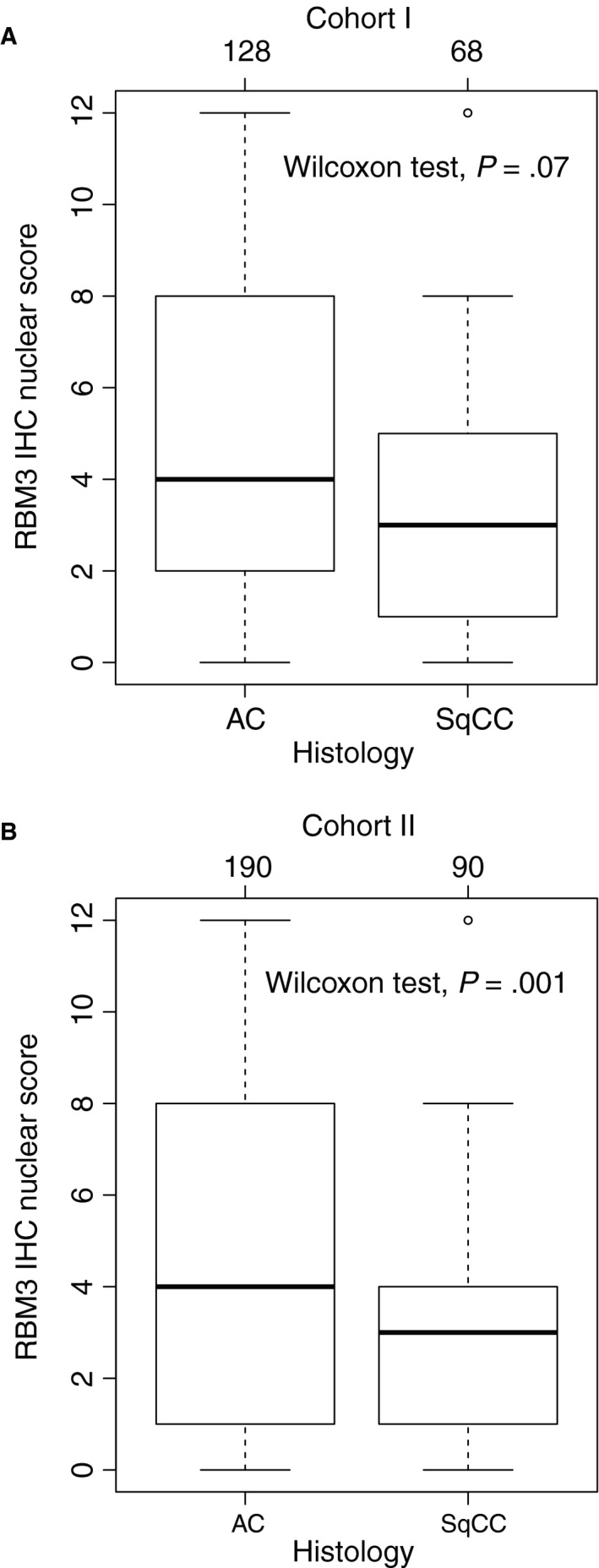
Comparison of RBM3 protein expression (nuclear score) among adenocarcinomas (AC) and squamous cell carcinomas (SqCC) for cohort I (A) and cohort II (B)

Immunohistochemical RBM3 protein expression could be evaluated in 209 (98%) of 213 cases in cohort I and in 301 (98%) of 306 cases in cohort II. There was no obvious heterogeneity between tissue cores. Representative microscopic images of RBM3 in lung cancer cases and benign lung tissue are shown in Figure S1. When comparing NS, AC had higher expression of RBM3 compared to SqCC, although statistically significant only in cohort II (Mann‐Whitney test, *P* = .07 and *P* = .001, Figure 1A,B). In cohort I, the cut‐off for AC (NS ≥8) resulted in 42 cases (33% of AC) classified as having a high RBM3 expression and the cut‐off for SqCC (NS ≥4) resulted in 32 cases (47% of SqCC) classified as having a high RBM3 expression. In cohort II, 73 AC cases (38% of AC) and 35 SqCC cases (39% of SqCC) had a high RBM3 expression.

In cohort II, AC cases with high RBM3 protein expression were older compared to AC cases with low expression (median 68 vs 66 years, Mann‐Whitney test, *P* = .02). Apart from this finding, there were no other associations between RBM3 protein expression (high/low) and age, gender, stage, growth pattern (AC only), smoking, adjuvant treatment, or WHO performance status (cohort II only) in either AC or SqCC analyzed separately in cohorts I and II.

When T and N stages were analyzed separately, low RBM3 was significantly associated with higher N stage in SqCC in cohort II only (*P* = .01). The relationship between the most prevalent mutations and RBM3 were analyzed in cohort II. In AC, RBM3 (high vs. low) was not associated with mutation in any of *CSMD3*, *EGFR*, *KRAS*, *LRP1B*, *MUC16*, *STK11*, or *TP53* (Fischer's exact test, *P* = .17‐0.65). Similarly, in SqCC, RBM3 was not associated with mutation in *CSMD3*, *KMT2D*, *LRP1B*, *MUC16*, *PIK3CA*, *TP53* (Fischer's exact test, *P* = .12‐1.0).

### 3.3 Association of RBM3 protein expression with outcome in NSCLC

#### 3.3.1 Cohort I

**Figure 2:**
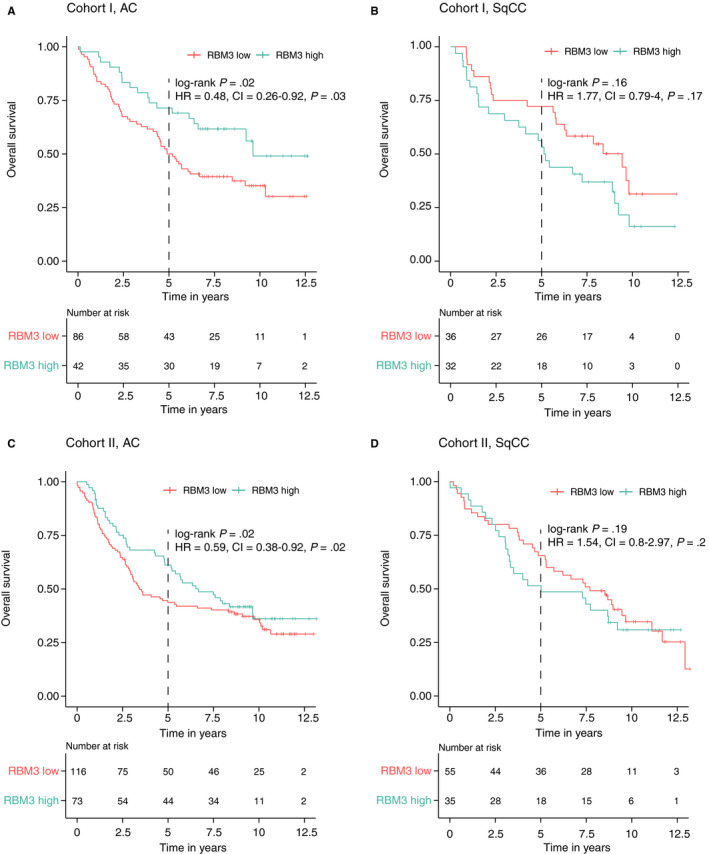
Prognostic value of RBM3 protein expression on overall survival in adenocarcinomas (AC) in cohort I (A), squamous cell carcinomas (SqCC) in cohort I (B), AC in cohort II (C) and SqCC in cohort II (D)

In the 5‐year OS analysis, high RBM3 expression was a favorable prognostic factor for AC (log‐rank test, *P* = .02, Figure 2A). The association of high RBM3 protein expression with improved prognosis in AC was significant in the univariable Cox proportional hazards regression model (HR 0.48, 95% CI 0.26‐0.92). RBM3 remained prognostic also in the multivariable model adjusted for stage, growth pattern, age, gender, smoking, and adjuvant treatment (HR 0.44, 95% CI 0.21‐0.90). Stage was the only other significant factor in the multivariable model (HR 2.47, 95% CI 1.60‐3.82). No relation between RBM3 expression and adjuvant treatment could be seen, see Data S1 and Figure S2.

In the RFI analysis, AC cases with high expression of RBM3 had a lower rate of recurrence, although not statistically significant (Figure S3A).

In SqCC, an opposite relationship between RBM3 expression and prognosis was observed, although not statistically significant in either OS analysis or RFI analysis (Figure 2B and Figure S3B).

Excluding the cases with stage IIIB‐IV did not significantly affect the results for AC or SqCC.

#### 3.3.2 Cohort II

In the validation cohort, RBM3 was confirmed as a favorable prognostic factor among AC in the 5‐year OS analysis (log‐rank test, *P* = .02, Figure 2C). RBM3 remained prognostic in the univariable Cox proportional hazards regression model (HR 0.59, 95% CI 0.38‐0.92, Figure 2C) but not in the multivariable model, adjusted for stage, growth pattern, age, gender, smoking, WHO performance status, and adjuvant treatment (HR 0.65, 95% CI 0.39‐1.10). Stage (HR 1.84, 95% CI 1.35‐2.51), WHO performance status (HR 1.95, 95% CI 1.28‐2.99) and growth pattern (HR 1.63, 95% CI 1.04‐2.53) were instead significant factors.

Regarding RFI, AC cases with high expression of RBM3 had a lower rate of recurrence (log‐rank test, *P* = .01, Figure S3C). Similar results were obtained in the univariable Cox proportional hazards regression model, where AC with high RBM3 protein expression had a decreased risk of recurrence (HR 0.53, 95% CI 0.32‐0.89) and this association remained significant in the multivariable model, adjusted for stage, growth pattern, age, gender, smoking, WHO performance status, and adjuvant treatment (HR 0.38, 95% CI 0.22‐0.74). Stage was the only other significant factor in the multivariable model (HR 1.73, 95% CI 1.21‐2.47).

In the 5‐year OS analysis for SqCC, an opposite relationship between RBM3 expression and prognosis was again observed (Figure 2D), although not statistically significant. Regarding RFI analysis, SqCC cases with a high expression of RBM3 had a significantly higher risk of recurrence (log‐rank test, *P* = .03, Figure S3D). We observed similar results in the Cox proportional hazards regression model where SqCC with high RBM3 protein expression had an increased risk of recurrence (HR 2.3, 95% CI 1.06‐4.97 in the univariable model and HR 2.57, 95% CI 1.11‐5.96 in the multivariable model adjusted for stage, age, gender, smoking, WHO performance status, and adjuvant treatment).

Excluding the cases with stage IIIB‐IV did not significantly affect the results for AC or SqCC.

### 3.4 Associations of RBM3 and Ki67 expression

**Figure 3:**
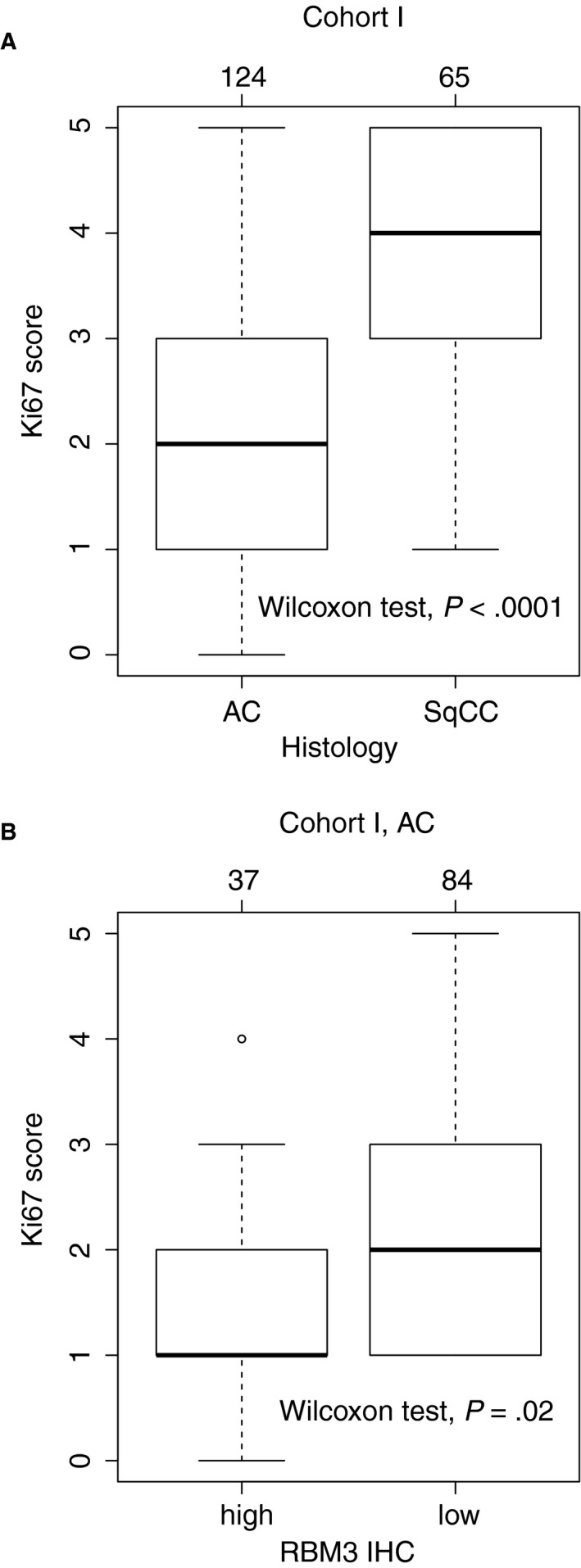
Protein expression of Ki67 among adenocarcinomas (AC) and squamous cell carcinomas (SqCC) in cohort I (A) and in relation to RBM3‐classification among AC in cohort I (B)

Protein expression of Ki67 was assessed with IHC staining in cohort I. AC cases had lower expression of Ki67 compared to SqCC cases (Mann‐Whitney test, *P* < .0001, Figure 3A). In AC, we found a negative correlation between Ki67 and RBM3 protein expression (Mann‐Whitney test, *P* = .02, Figure 3B). Microscopic images of Ki67 and RBM3 from the same AC cases are shown in Figure S4. For SqCC, no association between Ki67 and RBM3 could be demonstrated (data not shown).

### 3.5 RBM3 gene expression in cohort II

RNA‐sequencing data from both tumor and paired normal tissue was available from 19 patients but showed no difference in *RBM3* gene expression levels (Figure S5A).

AC had higher levels of *RBM3* mRNA than SqCC (Mann‐Whitney test, *P* < .0001, Figure S5B).

The correlation between *RBM3* gene expression and IHC classification was evaluated in 101 AC cases (38 classified as RBM3 IHC high and 63 as RBM3 IHC low) and 57 SqCC cases (25 classified as RBM3 IHC high and 32 as RBM3 IHC low). A correlation between *RBM3* gene expression and IHC classification was observed in both AC and SqCC samples (for AC: Mann‐Whitney test, *P* = .0002, Figure S5C; for SqCC: Mann‐Whitney test, *P* = .03, Figure S5D).

The prognostic value of *RBM3* gene expression in cohort II was evaluated separately in 103 AC cases and 58 SqCC cases. No prognostic value of *RBM3* mRNA levels was observed among AC or SqCC in the 5‐year OS analysis, regardless if high *RBM3* mRNA was defined as the upper ⅓ or the upper ½ of the samples.

### 3.6 RBM3 gene expression in publicly available datasets

To investigate the association of *RBM3* gene expression with patient outcome, we analyzed 2087 AC and 899 SqCC assembled from 13 and 8 reported studies, respectively.

For AC, when comparing the upper ⅓ of the samples to the lower ⅔, higher *RBM3* gene expression was associated with better patient outcome in two of the 13 datasets (Table 2). In a pooled analysis (excluding Lee et al due to different endpoint ^27^), higher *RBM3* gene expression was weakly associated with overall survival in AC (log‐rank test, *P* = .02 and Cox univariable analysis, HR 0.81, 95% CI 0.68‐0.97), although this result may be partly driven by the single large Shedden et al cohort ^28^ (Table 2). When classifying ½ of the samples in each dataset as having high *RBM3* expression, no association with patient outcome was seen in any of the datasets (Table 2).

**Table 2:** *RBM3* gene expression and association with clinical outcome in adenocarcinoma

Cohort	No. of cases	Outcome type	Cut‐off upper ⅓ Log‐rank test, *P*‐value	Cut‐off ½ Log‐rank test, *P*‐value
Beer et al 2002	86	OS	.90	.20
Bild et al 2006	58	OS	.74	.14
Lee et al 2008	63	DMFS	.48	.38
Shedden et al 2008	444	OS	**.004**	.17
Chitale et al 2009	102	OS	.63	.82
Tomida et al 2009	117	OS	.11	.40
Hou et al 2010	45	OS	.51	.72
Fouret et al 2012	103	OS	.74	.69
Okayama et al 2012	226	OS	.76	.31
CLCGP/NGM 2013	98	OS	.72	.38
Sato et al 2013	183	OS	.82	.82
Der et al 2014	127	OS	**.04**	.46
TCGA 2014	435	OS	.20	.06
Pooled cohorts^a^	2024	OS	**.02**	.07

Abbreviations: DMFS, distant metastasis‐free survivalOS, overall survival.

^a^ Excluding Lee et al.

For SqCC, no prognostic value of *RBM3* mRNA levels was seen in either of the public datasets or in a pooled analysis (Table S1).

## 4 DISCUSSION

RBM3 is a highly conserved stress‐induced mRNA‐binding protein involved in regulation of gene expression at posttranscriptional level, for example, by affecting mRNA stability and translation.^3^ Apart from protection against adverse conditions such as hypothermia, hypoxia, radiation and drugs, an important role for RBM3 has been shown in cell proliferation and neuronal development. Its exact role in tumorigenesis is not fully understood. Upregulation has been demonstrated in various cancers, and in addition to its importance in cell cycle progression and anti‐apoptotic functions, RBM3 has been linked to increased stem cell characteristics. A high expression of RBM3 has been linked to better prognosis in numerous studies on clinical tumor specimens,^4^, ^5^, ^6^, ^7^, ^8^, ^9^, ^10^, ^11^, ^12^, ^13^, ^14^, ^15^ suggestively through inhibition of tumor growth and dissemination, where influence of DNA damage checkpoint protein levels is one possible mechanism.^3^

In this study, we found a correlation between high RMB3 protein expression and improved outcome in AC, and the multivariable analyses primarily suggest that RBM3 is an independent prognostic marker in surgically treated lung AC, although statistical significance was not seen for OS in cohort II. In contrast, no clear association with patient outcome was found for *RBM3* gene expression levels. In SqCC the opposite relationship between RBM3 protein expression and prognosis was observed, although statistically significant only in RFI analysis for cohort II. Moreover for SqCC, the results should be considered with care due to the small number of cases.

In a recent study by Melling et al,^15^ high RBM3 protein expression was found to be a favorable prognostic factor in lung AC but not in SqCC, which is in line with our results. In Melling et al, however, multivariable analysis was not performed, which hindered assessment of the independent prognostic ability of RBM3. Also, a different antibody was used, and the classification procedure of samples into high/low was not clarified which makes direct comparisons with our study difficult.

Herein, we used a well‐validated monoclonal antibody for evaluation of RBM3 expression ^5^ and considered a combination of the nuclear fraction and intensity for the assessment of RBM3 staining that has been used in previous studies.^4^, ^5^, ^6^, ^7^, ^9^, ^10^, ^11^, ^12^, ^41^ We were able to test the consistency of the chosen cut‐offs through our discovery and validation strategy in two independent datasets; and by performing multivariable analysis, we could assess the independent prognostic value of RBM3. We used different cut‐offs for AC and SqCC since the two histologies had different levels of RBM3 protein expression, an approach to consider in future studies or if using assessment of RBM3 in a clinical setting.

We found a negative, albeit weak, correlation between expression of RBM3 and the proliferation marker Ki67 (assessed by IHC in cohort I). This was not unexpected, since Ki67 is a well‐known negative predictor of lung cancer survival. However, the correlation might also imply that the prognostic ability of RBM3 is linked to proliferation. A negative correlation between Ki67 and RBM3 has previously been observed in breast cancer (although not confirmed in malignant melanoma and upper gastrointestinal AC).^4^, ^10^, ^42^ SqCC had higher levels of Ki67 compared to AC in our study, which has been previously demonstrated by, for example, Warth et al,^43^ where they also found that Ki67 had an opposite relationship with patient survival in AC and SqCC, in line with our findings regarding RBM3 protein expression. The correlation between RBM3 and Ki67, the lower levels of RBM3 (and higher levels of Ki67) among SqCC compared to AC, and the potentially opposite relationship between RBM3 and prognosis in the two histologies merit further investigation.

Previous studies examining the prognostic role of *RBM3* gene expression levels in other types of cancer have generated conflicting results.^5^, ^16^ In our study, we found a statistically significant but small difference in *RBM3* mRNA levels between samples classified as RBM3 high and low (cohort II, samples with IHC and RNA sequencing data). However, we could not detect any prognostic value of *RBM3* gene expression levels.

Some limitations of the present study must be discussed. Although the inclusion of more than one center may better resemble the clinical setting and enable a validation strategy, separate analyses of the two cohorts rendered too small subgroups for some statistical analyses, where more cases would have been needed to fully evaluate the prognostic value of RBM3 in, for example, different lung cancer stages and in relation to adjuvant treatment. Investigation of RBM3 protein expression on TMAs instead of whole tumor sections is also a limitation. However, the TMAs had three (cohort I) or two (cohort II) cores, and there was generally a good concordance between the cores. Also, we could not update all cases to TNM 8, as, for example, a correct measure of invasion could not be determined in some of these older cases, why TNM 7 was kept. Furthermore, data were missing on treatment after recurrence, which might affect overall survival. Only few cases (0 of 52 tested AC in cohort I and 12 of 194 AC in cohort II) had both an *EGFR* mutation and recurrence, and these were equally distributed among high and low RBM3 expression, thus probably without impact on our main results.

In conclusion, the results from this study support RBM3 protein expression as a biomarker—suggestively independent—for improved prognosis in lung AC. Its value as a prognostic marker in lung cancer and its potential use in the clinical setting merit further investigation.

## CONFLICT OF INTERESTS

The authors declare no conflict of interest.

## AUTHOR CONTRIBUTIONS

KJ, MP, and HB designed the study. PM, JSSM, JB, and HB, and MJ, BN, KJ, JS, MP, and HB, created the cohorts. AS, L. LF., JI, JS, MP, and HB collected the data. AS, JS, MP, and HB analyzed the data. MJ, BN, and MU provided technical or other support. AS wrote the paper and all other authors reviewed the paper for its intellectual content.

## Supporting information

Supplementary Material

## Data Availability

The data that support the findings of this study are available on request from the corresponding author (HB). The data are not publicly available due to privacy/ethical restrictions.
