# Supplementary material for: Comprehensive analysis of RNA binding motif protein 3 (RBM3) in non‐small cell lung cancer
Source: Cancer Med. 2020 Jun 3;9(15):5609–19. doi: 10.1002/cam4.3149 (PMC7402820; doi:10.1002/cam4.3149)
Supplement: Supplementary file 1 — Supplementary Material [file CAM4-9-5609-s001.pdf]

Supporting information

Supplementary Text	page 2
Supplementary Table 1	page 3
Supplementary Figure 1	page 4
Supplementary Figure 2	page 7
Supplementary Figure 3	page 8
Supplementary Figure 4	page 9
Supplementary Figure 5	page 10
References (same numbers as in main paper)	page 11

Supplementary Text

RBM3 expression in paired metastases

In cohort II (“Uppsala NSCLC II cohort”), evaluation of RBM3 protein expression was possible in 36 paired metastases. The metastases consisted of concurrent surgically removed lymph node (n=28) or intrapulmonary metastases (n=5), or later discovered surgically removed metastases to the central nervous system (n=3). Of these, 27 were adenocarcinomas (AC) and 9 were squamous cell carcinomas (SqCC). For 24 (67%) of the 36 samples, both the primary tumor and the metastasis had the same dichotomized RBM3 score (high or low). In the remaining cases, RBM3 expression in the metastasis was equally often higher or lower than in the primary tumor.

Protein expression of RBM3 in metastases compared to primary tumors has been previously investigated in other types of cancer, with varying results. [8, 10, 16] The data of our study indicate a relatively good concordance regarding RBM3 expression between primary non-small cell lung carcinomas (NSCLC) and matched metastases. This suggests that in the metastatic setting, analysis of RBM3 protein expression in the primary tumor should be sufficient for previously surgically treated patients with NSCLC. However, the number of patients in our study is limited and further investigation is needed.

RBM3-expression in strata according to adjuvant treatment

We could not demonstrate any prognostic value of RBM3 expression in relation to adjuvant treatment among AC in either of the two cohorts. Since the number of cases receiving adjuvant treatment in each cohort was limited, a pooled analysis of AC from both cohorts I and II was performed (see Supplementary Figure 2 below), but the results remained unaffected. Likewise, analysis of the prognostic value of RBM3 expression according to adjuvant treatment among SqCC did not reveal any significant results, either in separate analyses of the two cohorts or in a pooled analysis (data not shown).

A connection between RBM3 expression and sensitivity to chemotherapy has been suggested, as *in vitro* studies have shown that downregulation of RBM3 through si-RNA confers resistance to chemotherapy. [5, 16] However, in our study, the association between RBM3 expression and prognosis among patients treated with adjuvant chemotherapy was similar to the non-treated group. Still, larger cohorts may be needed for further investigation of the matter.

Supplementary Table 1 *RBM3* gene expression and association with clinical outcome in squamous cell carcinoma.

Cohort	No. of cases	Outcome type	Cut-off upper ⅓ Log-rank test, p-value	Cut-off ½ Log-rank test, p-value
Bild et al. 2006	53	OS	0.65	0.5
Raponi et al. 2006	130	OS	0.08	0.14
Lee et al. 2008	75	DMFS	0.76	0.96
Hou et al. 2010	27	OS	0.44	0.10
CLCGP/NGM 2013	93	OS	0.61	0.3
Sato et al. 2013	80	OS	0.22	0.59
Der et al. 2014	43	OS	0.95	0.51
TCGA 2014	398	OS	0.19	0.68
Pooled cohorts [a]	824	OS	0.33	0.85

Abbreviations: OS, overall survival; DMFS, distant metastasis-free survival

[a] Excluding Lee et al.

Supplementary Figure 1 Microscopic images of immunohistochemical RBM3 staining in lung. **(A-B)** Non-malignant lung parenchyma and airway with chronic inflammation. Basal cells of bronchioles and submucous glands were consistently weakly positive while immune cells, vascular endothelial cells and reactive type II pneumocytes were inconsistently positive in the specimens. **(C-D)** Negative adenocarcinoma (AC). **(E-F)** Weakly positive AC. **(G-H)** Moderately positive AC. **(I-J)** Strongly positive AC. Note weak positivity in vascular endothelial cells in adjacent artery **(J)**. **(K-L)** Negative squamous cell carcinoma (SqCC). **(M-N)** Weakly positive SqCC. **(O-P)** Moderately positive SqCC. **(Q-R)** Strongly positive SqCC.

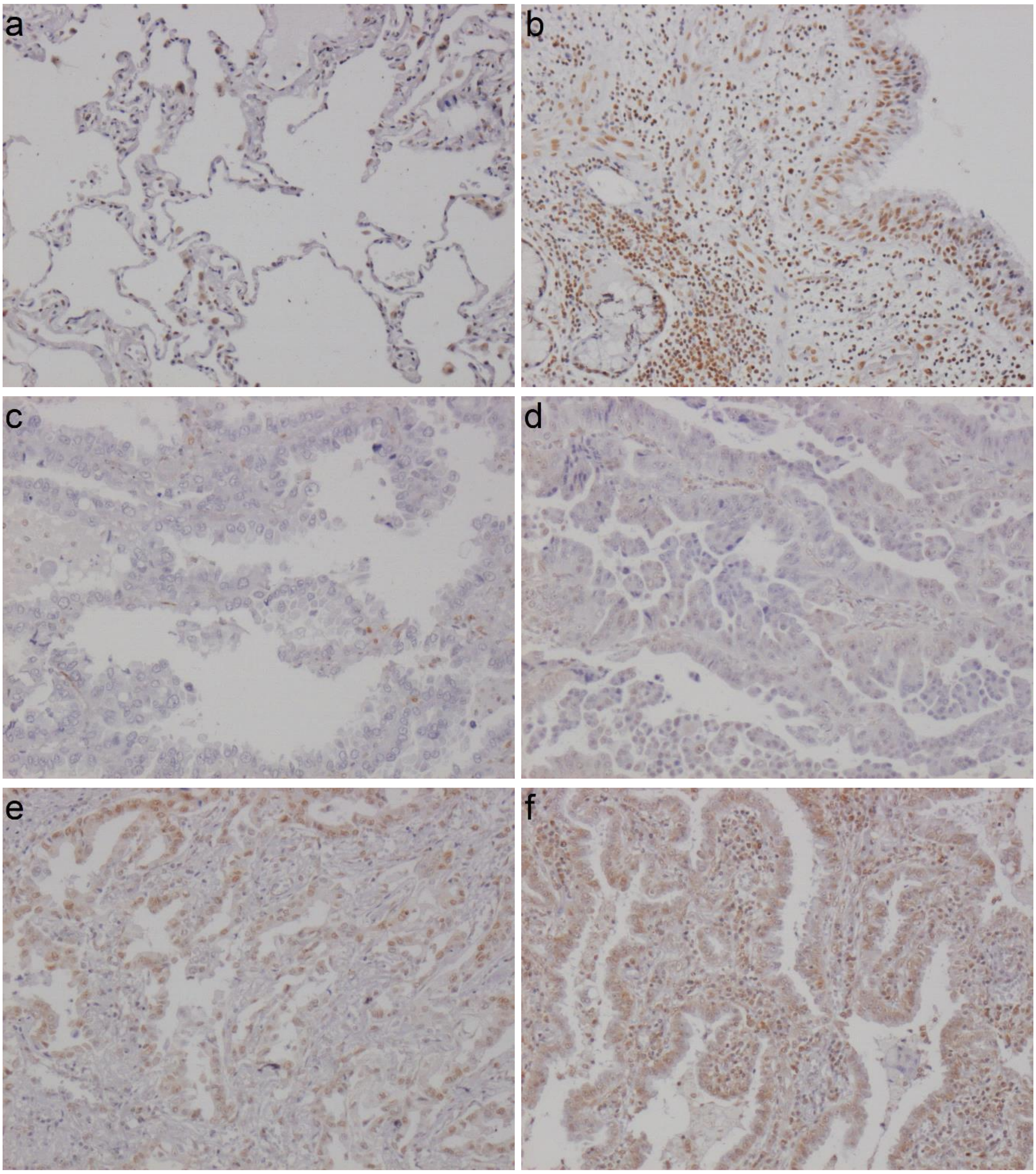

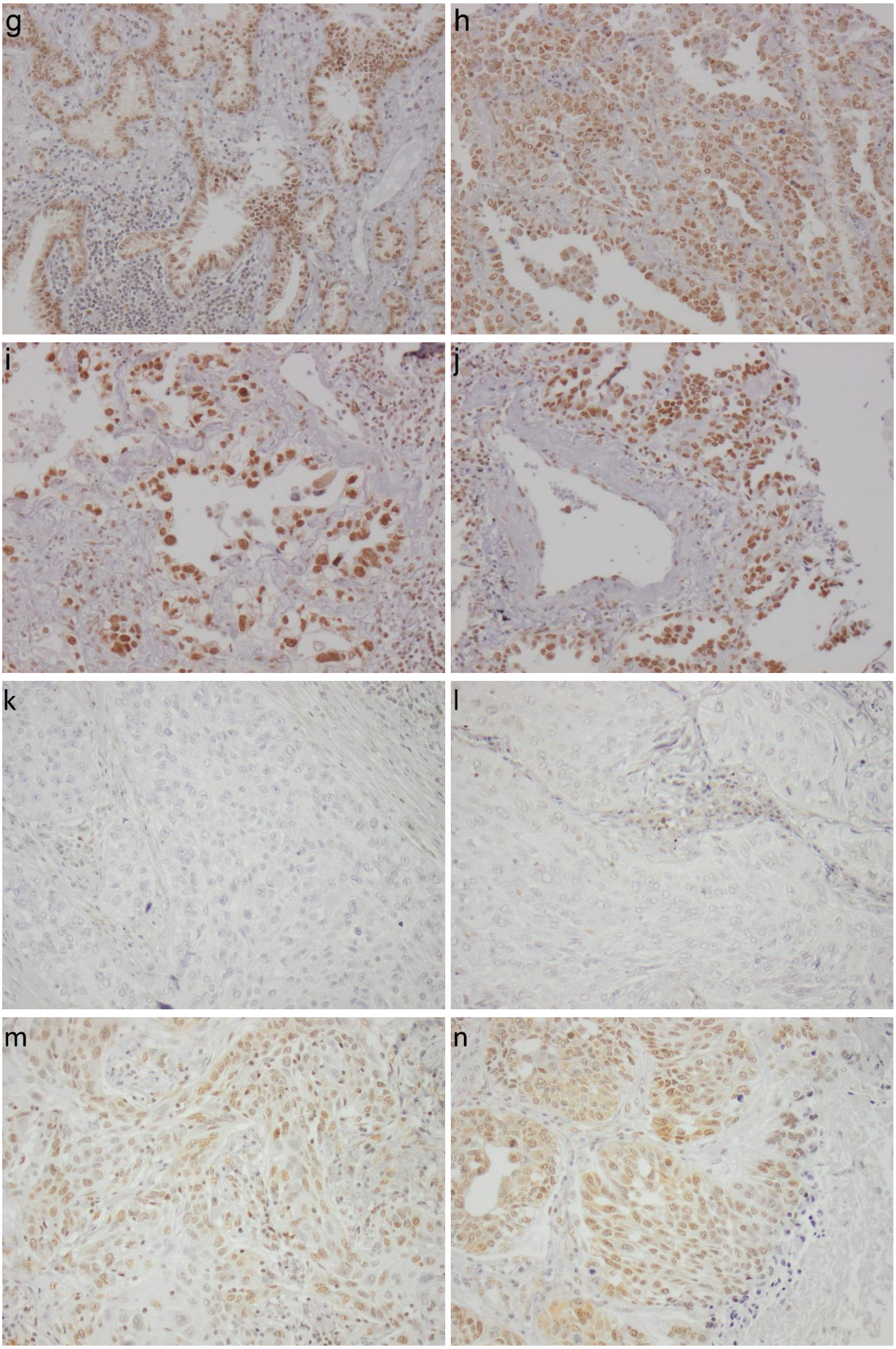
g
h
i
j
k
l
m
n

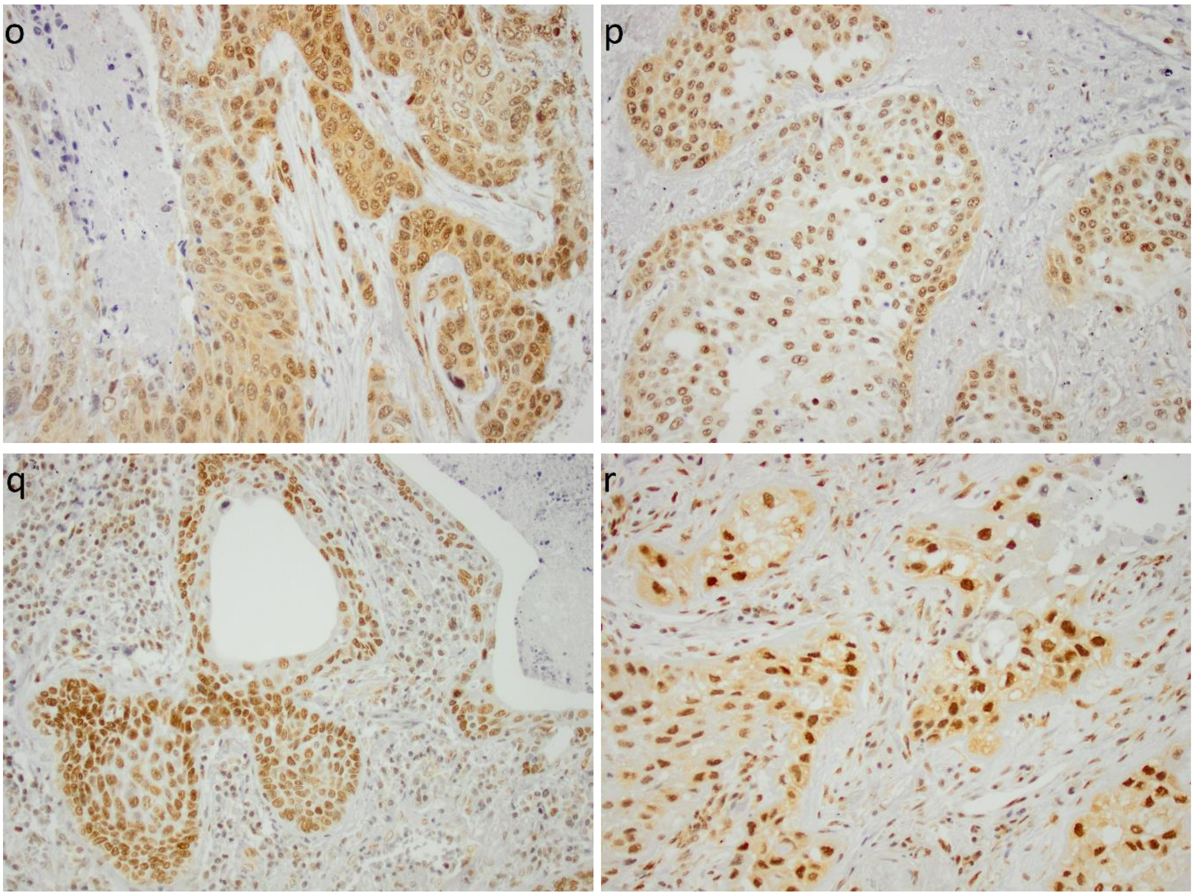
o
p
q
r

Supplementary Figure 2 Prognostic value of RBM3 protein expression on overall survival according to adjuvant treatment. Pooled analysis of adenocarcinomas (AC) in cohorts I and II.

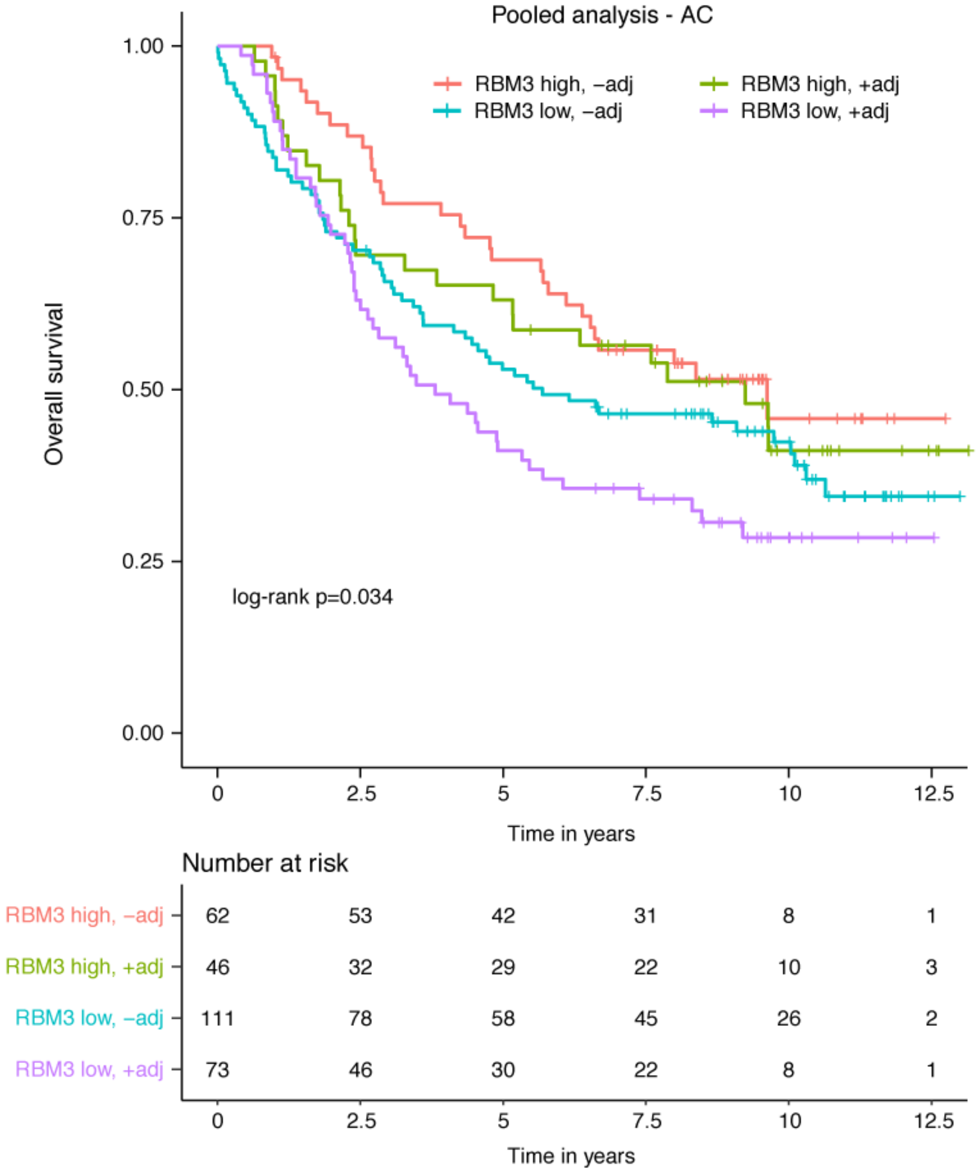

Supplementary Figure 3 Prognostic value of RBM3 protein expression on recurrence-free interval in adenocarcinomas (AC) in cohort I (**A**), squamous cell carcinomas (SqCC) in cohort I (**B**), AC in cohort II (**C**) and SqCC in cohort II (**D**).

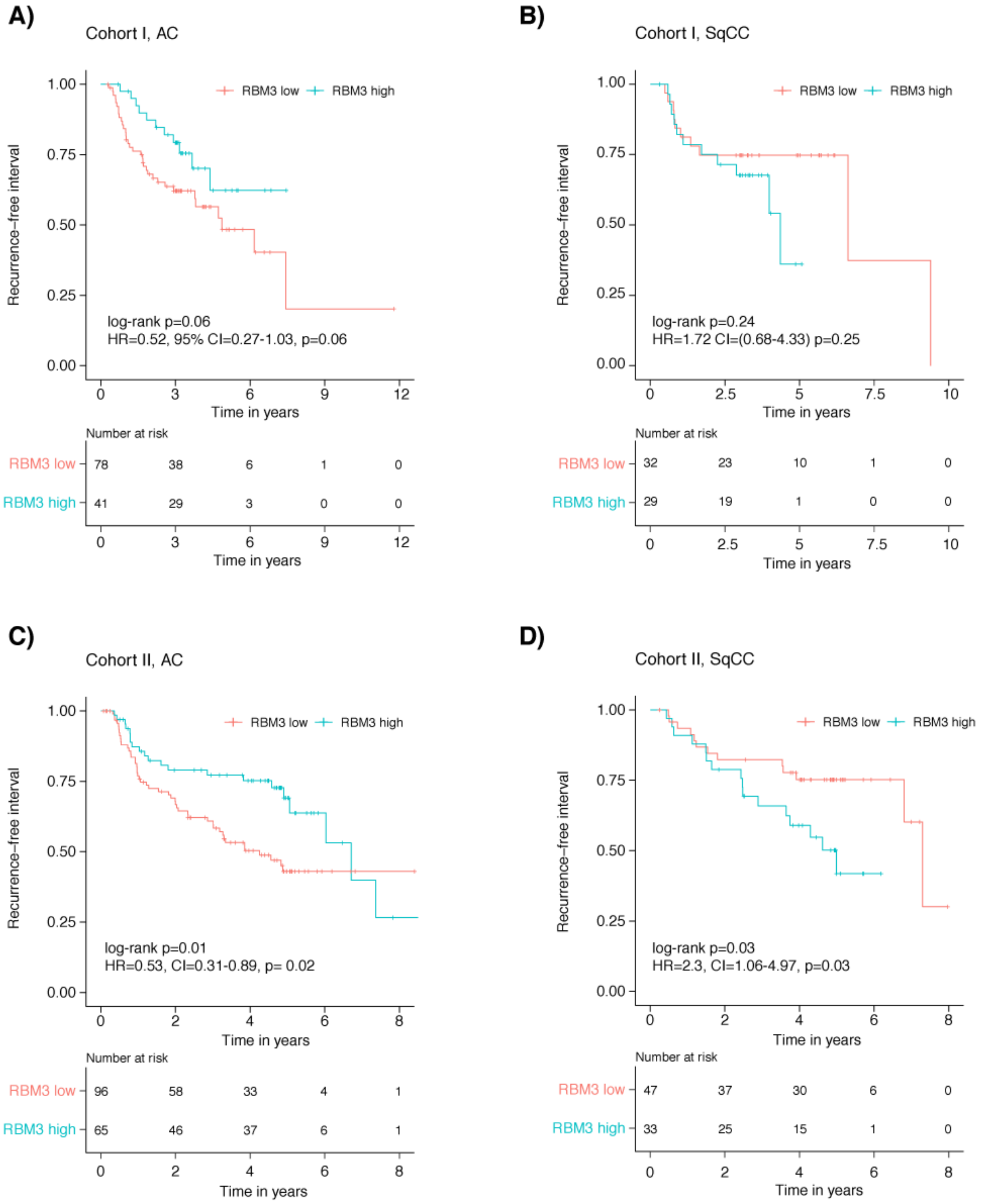

Supplementary Figure 4 Microscopic images of paired immunohistochemical RBM3 and Ki67 staining in lung adenocarcinomas illustrating the inverse relationship of the two markers. For each case, RBM3 and Ki67 is from the same tissue microarray core. **(A-B)** A case with low Ki67 **(A)** and high RBM3 **(B)**. **(C-D)** A case with moderate both Ki67 **(C)** and RBM3 **(D)**. **(E-F)** A case with high Ki67 **(E)** and low RBM3 **(F)**.

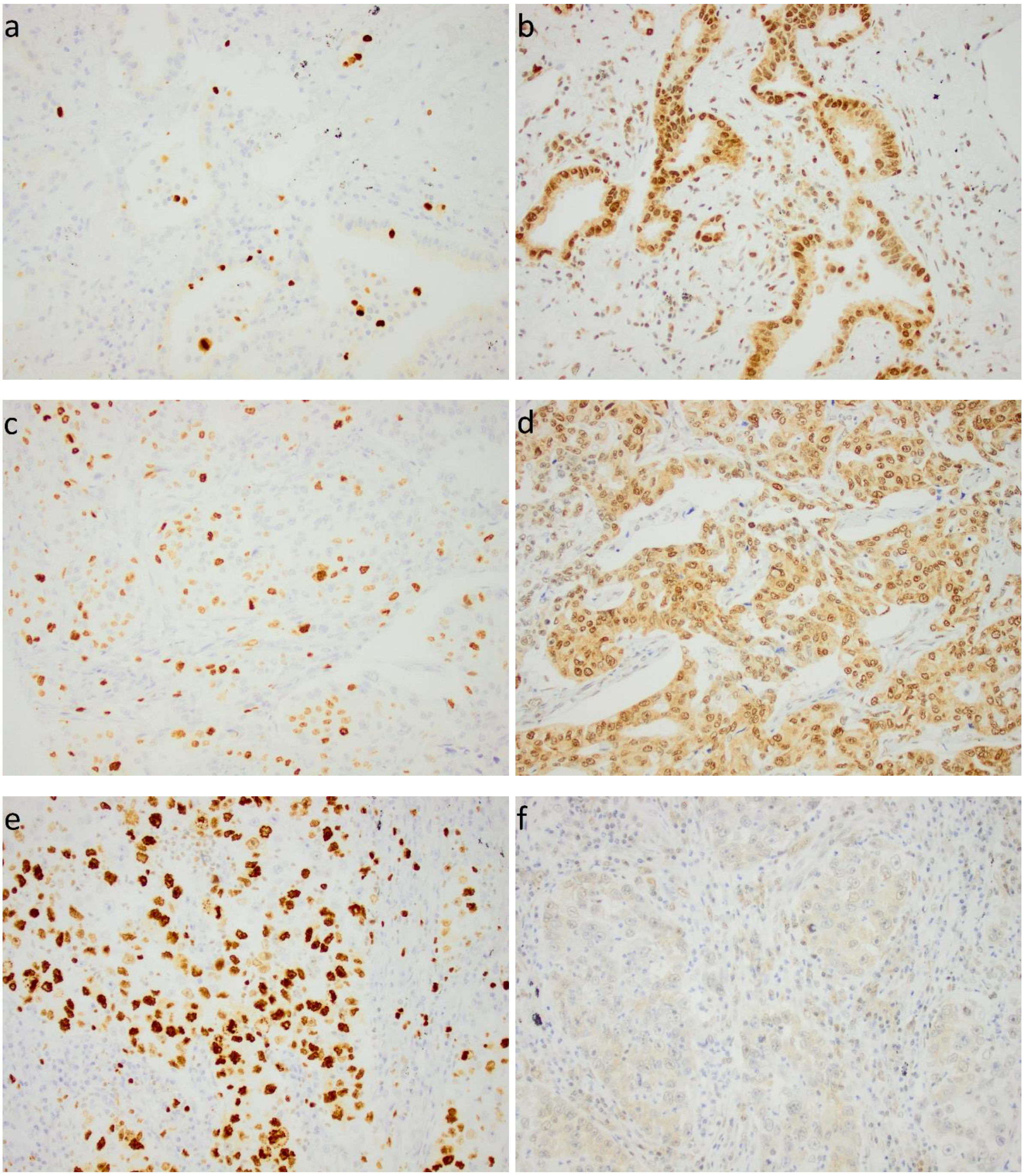

Supplementary Figure 5 *RBM3* gene expression in cohort II. Comparison of expression in tumor samples and paired normal tissue (**A**), in adenocarcinomas (AC) and squamous cell carcinomas (SqCC) (**B**), and in relation to RBM3-classification among AC (**C**) and SqCC (**D**).

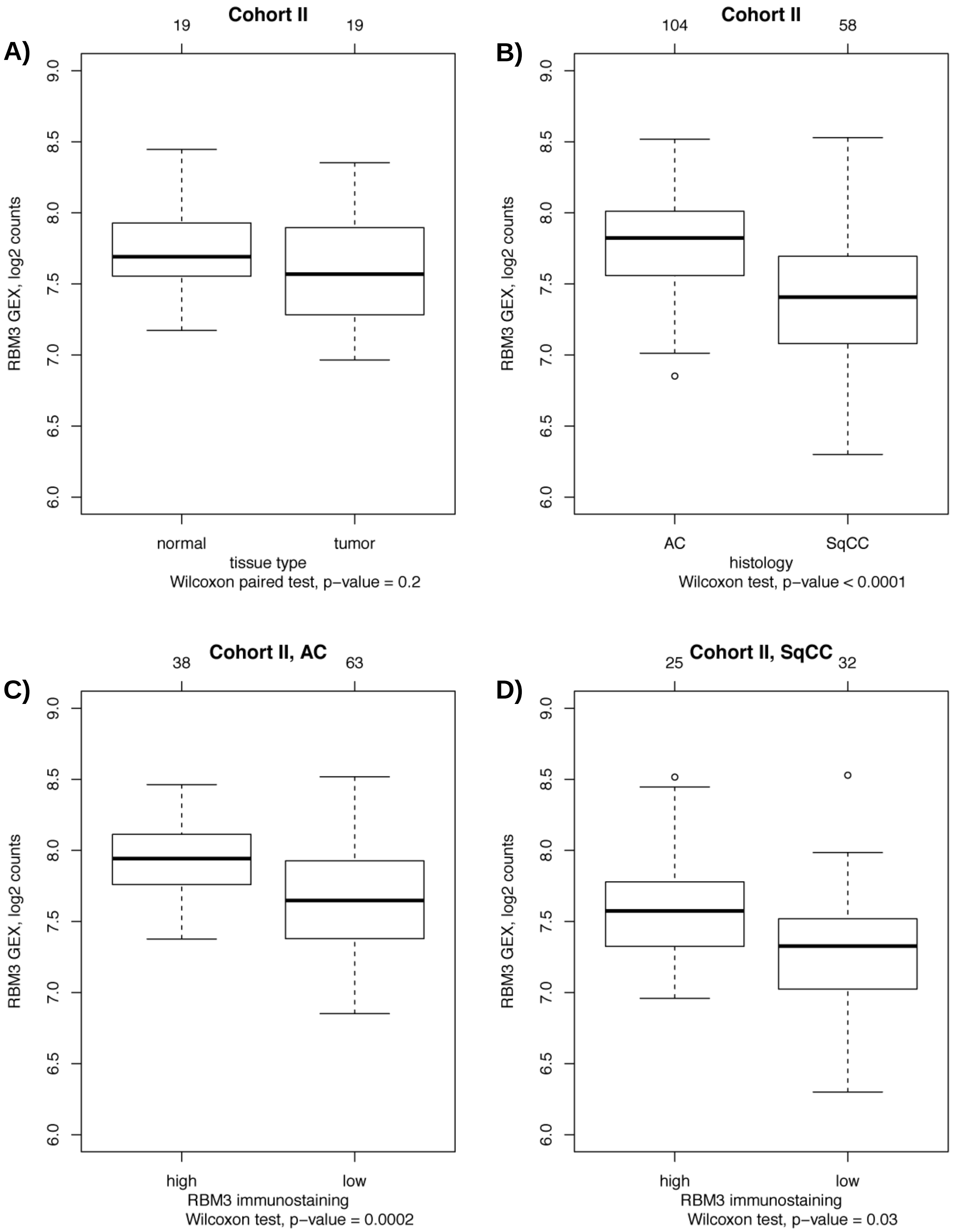

References

5. Ehlen A, Brennan DJ, Nodin B, et al. Expression of the RNA-binding protein RBM3 is associated with a favourable prognosis and cisplatin sensitivity in epithelial ovarian cancer. J Transl Med 2010; 8: 78.

8. Jonsson L, Bergman J, Nodin B, et al. Low RBM3 protein expression correlates with tumour progression and poor prognosis in malignant melanoma: an analysis of 215 cases from the Malmo Diet and Cancer Study. J Transl Med 2011; 9: 114.

10. Jonsson L, Hedner C, Gaber A, et al. High expression of RNA-binding motif protein 3 in esophageal and gastric adenocarcinoma correlates with intestinal metaplasia-associated tumours and independently predicts a reduced risk of recurrence and death. Biomark Res 2014; 2: 11.

16. Karnevi E, Dror LB, Mardinoglu A, et al. Translational study reveals a two-faced role of RBM3 in pancreatic cancer and suggests its potential value as a biomarker for improved patient stratification. Oncotarget 2018; 9: 6188-6200.
